# ECG abnormality and predictor of new‐onset atrial fibrillation in hypertension and diabetes mellitus population: An observational analytic study from cardiovascular outpatient clinic at a National Cardiovascular Center in Indonesia

**DOI:** 10.1002/joa3.12938

**Published:** 2023-10-09

**Authors:** Irawati Hajar Kikuko, Rerdin Julario, A’rofah Nurlina Puspitasari, Ryan Enast Intan, Yusuf Azmi, Fahrun Nisa’i Fatimah, Cornelia Ghea Savitri, Dwika Rasyid Firmanda, Lidya Pertiwi Suhandoko, Atikah S. Fildzah Dini

**Affiliations:** ^1^ Department of Cardiology and Vascular Medicine, Faculty of Medicine Airlangga University, Dr. Soetomo General Hospital Surabaya Indonesia

**Keywords:** arrhythmia, diabetes mellitus, hypertension, new‐onset atrial fibrillation

## Abstract

**Background:**

Population‐based studies have also found that diabetes mellitus (DM) and hypertension (HT) are independent risk factors for atrial fibrillation (AF). However, less is known about new‐onset atrial fibrillation (NOAF) risk factors and its correlation with DM and HT. The aim of this study was to determine the prevalence and pattern of ECG abnormalities, and the predictor of NOAF in patients with HT and DM.

**Methods:**

This cross‐sectional study was conducted at a tertiary hospital from May until December 2021. All medical record data from outpatients who had both diagnoses HT and DM were included in this study. Data from patients with unstable hemodynamics and lack of complete medical record data were excluded. Then, patient history, medical records, ECG, and laboratory information were reviewed.

**Results:**

There were 162 patients included in this study. Arrhythmia was found in 14.2% of the population, with new‐onset AF (NOAF) as the most common finding with 8.6% incidence, followed by PVC (3.1%) and PAC (2.5%). Bivariate analysis showed that valvular heart disease, random blood glucose, LVEF, and infection status were associated with a higher incidence of NOA. Model from multivariate logistic regression showed that valvular heart disease and random blood glucose level were independently correlated with NOAF (*p* = .009).

**Conclusion:**

It can be concluded that random blood glucose level at a certain point and valvular heart disease can be used as a risk predictor of NOAF in the hypertension population with concomitant DM.

## Graphical abstract

Arrhythmia was found in 14.2% of the population, with new‐onset AF (NOAF) as the most common finding with 8.6% incidence, followed by PVC (3.1%) and PAC (2.5%). Model from multivariate logistic regression showed that valvular heart disease and random blood glucose level were independently correlated with NOAF.

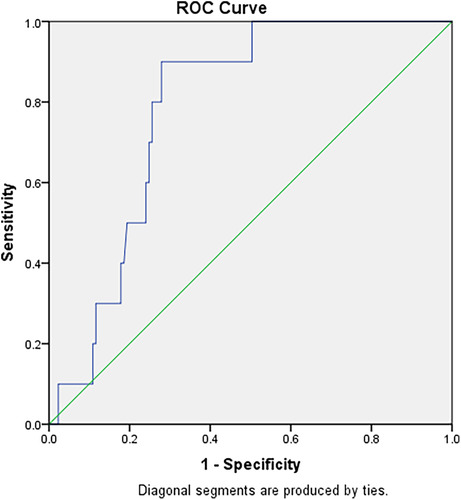

## 1 INTRODUCTION

Hypertension and diabetes mellitus (DM) are common chronic diseases that affect a large proportion of the population worldwide. This disease is considered as one of the main risk factors for cardiovascular disease. The 2016 WHO (World Health Organization) report states that hypertension affects 22% of the world's population, and 36% in Southeast Asia. The Indonesian Basic Health Research (Riskesdas) in 2018 showed the prevalence of hypertension was 34.1%.^1^ A report from the International Diabetes Federation (IDF) 2017 predicts the number of people with DM in the world is 425 million, while in Southeast Asia it is around 82 million. The 2018 Riskesdas results report shows that the average prevalence of DM in each province of Indonesia based on a doctor's diagnosis from a population aged 15 years in 2018 reached 2%. Meanwhile, the prevalence of DM based on blood tests from residents aged 15 years with the 2015 Perkeni consensus reached 10.9% in 2018.^2^

Research in Japan conducted by the Funagata, Ohasama, and Toyama et al. found that there were 20% of patients suffering from diabetes mellitus in hypertensive patients. The Strong Heart Study, with study subjects in Arizona, reported a 53.7% higher rate of diabetes mellitus in hypertensive patients. Patients suffering from hypertension with diabetes mellitus have a five times higher risk of CVD than those with hypertension alone, this is similar to the results of a study conducted by the UK Prospective diabetes, namely an increase in macrovascular and microvascular complications with HR 1.12 (*p* < .001) per 10 mm Hg for every increase in systolic pressure.^3^

The higher the incidence of CVD, the heart screening using an ECG is very important. ECG screening can not only identify CVD but can also determine the risk of future CVD events. The American Diabetes Association (ADA), the European Society of Cardiology, and the European Association for the Guideline for the Study of Diabetes (ESC/EASD) recommend routinely performing a resting ECG in patients with type 2 diabetes and hypertension or suspected CVD, without specifying a specific time interval.^4^ One of the most important clinical findings in resting ECG screening is detection of arrhythmia. The most common type of arrhythmia in the world is atrial fibrillation (AF). The prevalence of AF is expected to more than double in the next 50 years as the population ages 1 At the same time, diabetes has become a pandemic disease in both the developed and developing worlds. Chronic AF development is influenced by many factors, and the mechanism is unknown; however, there is emerging evidence linking AF and diabetes mellitus (DM), in which they share common risk factors such as hypertension, atherosclerosis, and obesity. Population‐based studies have also found that diabetes and hypertension are independent risk factors for atrial fibrillation.^5^ However, less is known about new‐onset atrial fibrillation (NOAF) risk factor and its correlation with diabetes and hypertension. The purpose of this study was to determine the prevalence and pattern of ECG abnormalities, and the predictor of new‐onset atrial fibrillation (NOAF) in patients with hypertension and diabetes mellitus who were treated at tertiary hospital in Indonesia.

## 2 METHODS

**FIGURE 1:**
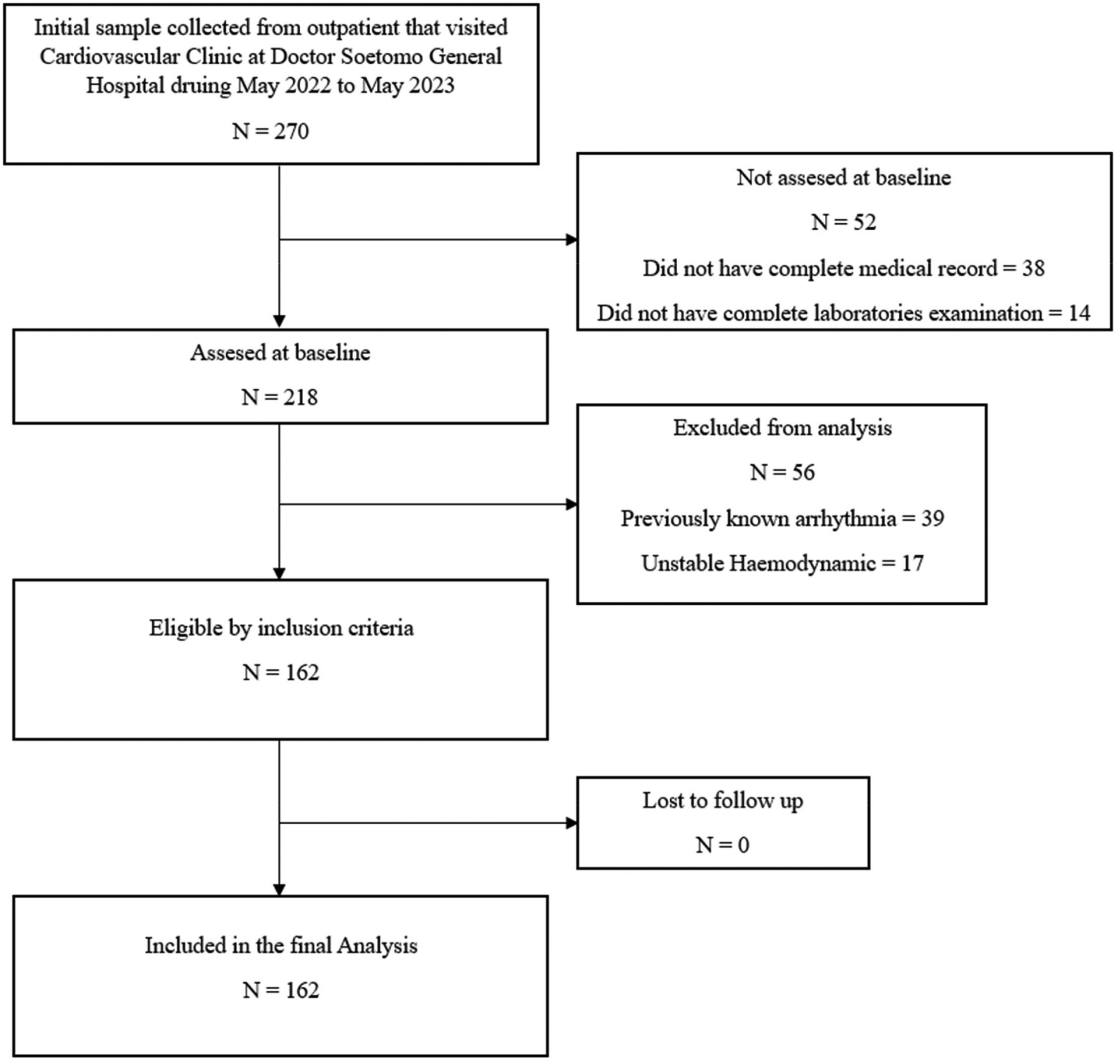
Study flow of this study. The initial sample of this study was 270 patients collected from outpatients who visited Cardiovascular Clinic at Doctor Soetomo General Hospital, Surabaya from May 2022 to May 2023. Then 108 samples are excluded from the analysis because of some reasons (not complete medical record = 38; not complete laboratory examination = 14; previously known arrhythmia = 39; unstable hemodynamics = 17). At the final analysis of the study, the number of patients who are included in this study was 162 patients.

This was a descriptive observational study using cross‐sectional design, conducted at Dr. Soetomo General Hospital, Surabaya, Indonesia, one of the biggest and main referral hospitals in Indonesia. The study was conducted from May 2022 to May 2023. Minimum of total sample in this study was 156 patients, based on sample size formula calculation by Kelsey et al.^6^ Inclusion criteria for this study was all adult patients with hypertension and type 2 diabetes who visited the cardiovascular outpatient clinic who has complete medical record data, ECG, and laboratory, without any known arrhythmia previously. Meanwhile exclusion criteria were patients with previously known arrhythmia such as atrial fibrillation, unstable hemodynamic and lack of complete medical record data were excluded. Study flow was described in Figure 1 that shows the sample selection process.

Diagnosis of hypertension in this study is based on ICD 10 di medical records (I10.XX), meanwhile hypertension category is based on ESC 2018 classification, with systolic blood pressure (SBP) ≥140 mm Hg and/or diastolic blood pressure (DBP) ≥90 mm Hg as HT Stage 1, and SBP ≥160 mm Hg and/or DBP ≥100 mm Hg as HT stage 2. Meanwhile, type 2 diabetes diagnosis is based on secondary data on medical records from ICD 10 code E11.XX. All the patient's medical record data who had both of the diagnosis (HT and DM) were included in the consecutive sampling.

Patient history, medical records, and laboratory information were reviewed to obtain data on patient, which include age, gender, history of diabetes mellitus, HTN, duration of HTN and diabetes mellitus, history of CVD and other comorbidities (lung disease and renal disease), and objective data which include heart rate, blood pressure, and random blood glucose (RBF) on the day of outpatient clinic visit. Patients were classified as having cardiovascular disease (CVD) if there was a history or review of previous medical records that revealed that the patient had a history of cerebrovascular disease (CVA/TIA), coronary artery disease (CAD), or peripheral vascular disease.

Information that might reveal the participants' identities was withheld. The Declaration of Helsinki was adhered to in this investigation. Since our study used secondary data and was an observational analytical study, no ethical approval was required.

Every patient underwent a 12‐lead resting ECG test. The following parameters on all ECGs were examined by a certified cardiologist: heart rhythm, heart rate, frontal axis, chamber dilatation, coronary abnormalities, and arrhythmia. New‐onset atrial fibrillation (NOAF) was defined as the first or new diagnosis of detectable episodes of AF, whether symptomatic or not. Patients who have history of AF before or with long‐standing AF or paroxysmal AF previously were excluded. The aforementioned variables were examined and manually measured. The definition used for this study will be given in Table S1. Data are presented in frequency and percentage. The primary outcome of this study was detection and predictor of new‐onset atrial fibrillation.

For the analytical process, all statistical analyses were performed using SPSS 25.0 (IBM Corp.). For descriptive analysis, continuous data are given as mean ± standard deviation (SD) or median (interquartile range [IQR]) depending on the distribution of the data, whereas categorical data are given as *n* (%). One‐Sample Kolmogorov‐Smirnov was used to assess the data distribution. To analyze the relationship between the independent variable and the NOAF as the dependent variable, bivariate analysis with chi‐square test was used to analyze categorical data, and independent *t*‐test or Mann‐Whitney *U*‐test was used for continuous data. Variables with *p* value <.25 from bivariate analysis were then included in multivariate logistic regression analysis if they were eligible. *p* value <.05 was considered statistically significant. All statistical analyzes were performed using SPSS for Windows version 21.0 (IBM Corp.).

## 3 RESULTS

There were total of 162 patients who met inclusion criteria in this study. Mean age was 59.5 ± 9.8 years old (Table 1). Majority of the patients were male (53.7%). The age distribution of hypertension cases with diabetes mellitus in this study occurred the most in the age range of 60–69 years with 63 patients (38.8% of the overall population), meanwhile, the least cases of hypertension with diabetes mellitus occurred in the age range of less than 40 years with only four patients (2.46%). The mean systolic and diastolic blood pressure values were 147.6 ± 28.2 mm Hg and 85.73 ± 17.8 mm Hg, respectively. The mean random blood glucose on our population was 162 ± 77 g/dL. Heart failure occurred in 27% of the population, meanwhile coronary artery disease was found in 65.43% of the population, with majority of single vessel disease (25.31%), and valvular heart disease found in 22.84% of the population. Other comorbidities include chronic kidney disease (CKD) (17.90%), cerebrovascular disease (CVD) (7.41%), anemia (6.79%), infection (5.00%), and lung disease (4.32%). For the treatment of hypertension, the most common used medication in this study was calcium channel blocker (CCB) (60.49%), followed by ace inhibitor/Angiotensin receptor blocker (ARB) (58.64%), and beta blocker (44.44%). For laboratory parameter, electrolyte imbalance was found in 11.73% of the population, with hyperkalemia and hypokalemia with 4.32% and 9.00% of the population, respectively. From baseline characteristics, there were no significant difference between controlled blood glucose (<180) and uncontrolled blood glucose (>180), except of electrolyte imbalance and the use of insulin treatment. (*p* < .05).

**TABLE 1:** Baseline characteristic of study population.

Variables	Total	Blood glucose < 180	Blood glucose > 180	*p* value
*N*	162 (100%)	117 (72.3%)	45 (27.7%)	
Sex *n* (%)				
Male	87 (53.70%)	61 (52.14%)	27 (60.0%)	.312
Female	74 (45.68%)	56 (47.86%)	18 (40.0%)	
Age ± SD (years)	59.5 ± 9.8	59.9 ± 10.4	58.4 ± 8.6	.713
Systolic BP (mean)	147.6 ± 28.2	144.8 ± 20.4	149.8 ± 29.0	.374
Diastolic BP (mean)	85.73 ± 17.8	84.5+ 16.7	86.3 ± 13.4	.132
Controlled HT	89 (55.00%)	64 (54.70%)	25 (56.00%)	.278
HT stage I	44 (27.00%)	34 (29.06%)	10 (22.00%)	
HT stage II	24 (14.81%)	18 (15.38%)	6 (13.33%)	
HT stage III	5 (3.09%)	2 (1.71%)	3 (6.67%)	
Comorbidities				
Heart failure	44 (27.00%)	31 (26.50%)	10 (22.00%)	.464
EF < 40%	27 (16.67%)	22 (18.80%)	5 (12.00%)	.215
EF >40%	135 (83.33%)	95 (81.20%)	40 (88.00%)	
Valvular heart disease	37 (22.84%)	26 (22.22%)	11 (24.00%)	.868
Coronary artery disease	106 (65.43%)	77 (65.81%)	29 (65.00%)	.637
Single vessel disease	41 (25.31%)	32 (27.35%)	9 (19.00%)	
Double vessel disease	30 (18.52%)	21 (17.95%)	8 (17.00%)	
Three vessel disease	38 (23.46%)	25 (21.37%)	13 (29.00%)	
Cerebrovascular disease	12 (7.41%)	7 (5.98%)	5 (10.00%)	.397
Ischemic stroke	8 (5.00%)	5 (4.27%)	3 (7.00%)	
Hemorrhage stroke	2 (1.23%)	1 (0.85%)	1 (3.00%)	
Chronic kidney disease	29 (17.90%)	18 (15.38%)	11 (24.00%)	.245
Anemia	11 (6.79%)	6 (5.13%)	5 (10.00%)	.272
Infection	8 (5.00%)	5 (4.27%)	3 (7.00%)	.512
Lung disease	7 (4.32%)	6 (5.13%)	1 (3.0.00%)	.751
Treatment				
ACE inhibitor/ARB	95 (58.64%)	66 (56.41%)	29 (65.00%)	.412
Calcium channel blocker	98 (60.49%)	71 (60.68%)	27 (59.00%)	.855
Beta blocker	72 (44.44%)	59 (50.43%)	13 (29.00%)	.165
Insulin	40 (24.69%)	23 (19.66%)	17 (37.00%)	**.036**
Oral antidiabetic agent	54 (33.33%)	35 (29.91%)	19 (42.00%)	.217
Laboratory RBG (mean) (mg/dL)	162 ± 77	131 ± 67	247 ± 71	.000
Electrolyte imbalance	19 (11.73%)	9 (7.69%)	10 (22.00%)	**.029**
Hyperkalemia	7 (4.32%)	4 (3.42%)	3 (7.00%)	.430
Hypokalemia	15 (9.00%)	7 (5.98%)	8 (17.00%)	.057

Abbreviations: ACE‐I, Angiotensin‐converting enzyme inhibitor; ARB, Angiotensin receptor blocker; BP, blood pressure; EF, ejection fraction; HT, hypertension; RBG, random blood glucose; SD, standard deviation.

Resting 12‐lead ECG examination showed that the mean heart rate was 83.2 ± 22.1 beats per minute, mean PR interval was 154.6 ± 27.2 ms, mean QRS duration 85.4 ± 15.9 ms. About 15% of the population were having tachycardia or bradycardia. From frontal axis, 6.8% of the population had left axis deviation (LAD), meanwhile from horizontal axis abnormality, majority (32.1%) had clockwise rotation (CWR). At least one ECG abnormality was found in 146 (90.12%) patients. Around 29% of the population also has coronary abnormality, with old myocardial infarction as the most frequent (20.90%). From chamber dimension, the most common finding was left ventricular hypertrophy (LVH) with 11.70%. As for the main outcome, arrhythmia was found in 14.2% of the population, with new‐onset AF (NOAF) as the most common finding with 8.6% incidence, followed by PVC (3.1%) and PAC (2.5%) (Table 2).

**TABLE 2:** ECG abnormalities.

Abnormalities	*N* = 162
	*n* (%)
Rate	
Normal	110 (67.9%)
Tachycardia	24 (14.8%)
Bradycardia	25 (15.5%)
Axis	
Left axis deviation	11 (6.8%)
Right axis deviation	3 (1.8%)
CWR	52 (32.1%)
CCWR	29 (18%)
Coronary abnormality	
Old myocardial infarct	34 (20.9%)
QS pattern	13 (8.1%)
Chamber dilatation	
Left Atrial Enlargement	6 (3.7%)
Left Ventricular Hypertrophy	19 (11.7%)
Right Ventricular Hypertrophy	1 (0.6%)
Right Atrial Enlargement	1 (0.6%)
Conduction abnormality	
LBBB	9 5.5%)
RBBB	2 (1.2%)
Arrhythmia	
Premature Ventricular Contraction	5 (3.1%)
Premature Atrial Contraction	4 (2.5%)
New‐onset atrial fibrillation	14 (8.6%)

Abbreviations: CCWR, counter clockwise rotation; CWR, clockwise rotation; ECG, electrocardiogram; LBBB, left bundle branch block; RBBB, right bundle branch block.

We continued to analyze the predictor of NOAF incidence from hypertensive patients who did univariate and multivariate logistic regression analysis. Variables which *p* value <.25 in univariate analysis were included in the multivariate logistic regression model. Univariate analysis showed that valvular heart disease, random blood glucose, LVEF, and infection status were associated with a higher incidence of NOAF. Model from multivariate logistic regression showed that valvular heart disease and random blood glucose level were independently correlated with NOAF (Table 3), with *p* model = .001, and good‐fitness of the data (Hosmer and Lemeshow test *p* = .474). The new model explained 47.2% (Nagelkerke *R* ^2^) of the variance and correctly re‐classified 96.1% of cases.

**TABLE 3:** Univariate and multivariate analysis of new‐onset atrial fibrillation (NOAF) incidence in hypertension and diabetic patients.

Variable	Univariate analysis			Multivariate analysis		
	HR	95% CI	*p*	HR	95% CI	*p*
Male	1.51	0.27–8.52	.642			
Age	1.05	0.96–1.14	.330			
Coronary artery disease	0.49	0.05–4.47	.531			
CVD	0.87	0.11–6.87	.893			
Heart failure	0.84	0.03–22.12	.918			
Anemia	1.31	0.10–17.14	.837			
Infection	9.87	0.39–24.58	.165	9.01	0.74–90.19	.085
Valvular heart disease	6.23	1.21–31.96	.028	4.95	1.25–19.65	.023
Electrolyte imbalance	0.565	0.043–7.51	.666			
Random blood glucose	1.008	1.00–1.02	.046	1.008	1.001–1.014	.034
Systolic blood pressure	0.98	0.93–1.04	.601			
Diastolic blood pressure	0.99	0.94–1.06	.959			
Resting Heart rate	0.72	0.34–1.78	.367			
% LVEF	3.25	0.62–16.92	.160	1.96	0.77–5.06	.162

Abbreviations: CI, confidence interval; CVD, cerebrovascular disease; HR, heart rate; LVEF, left ventricular ejection fraction; NOAF, new‐onset atrial fibrillation.

**FIGURE 2:**
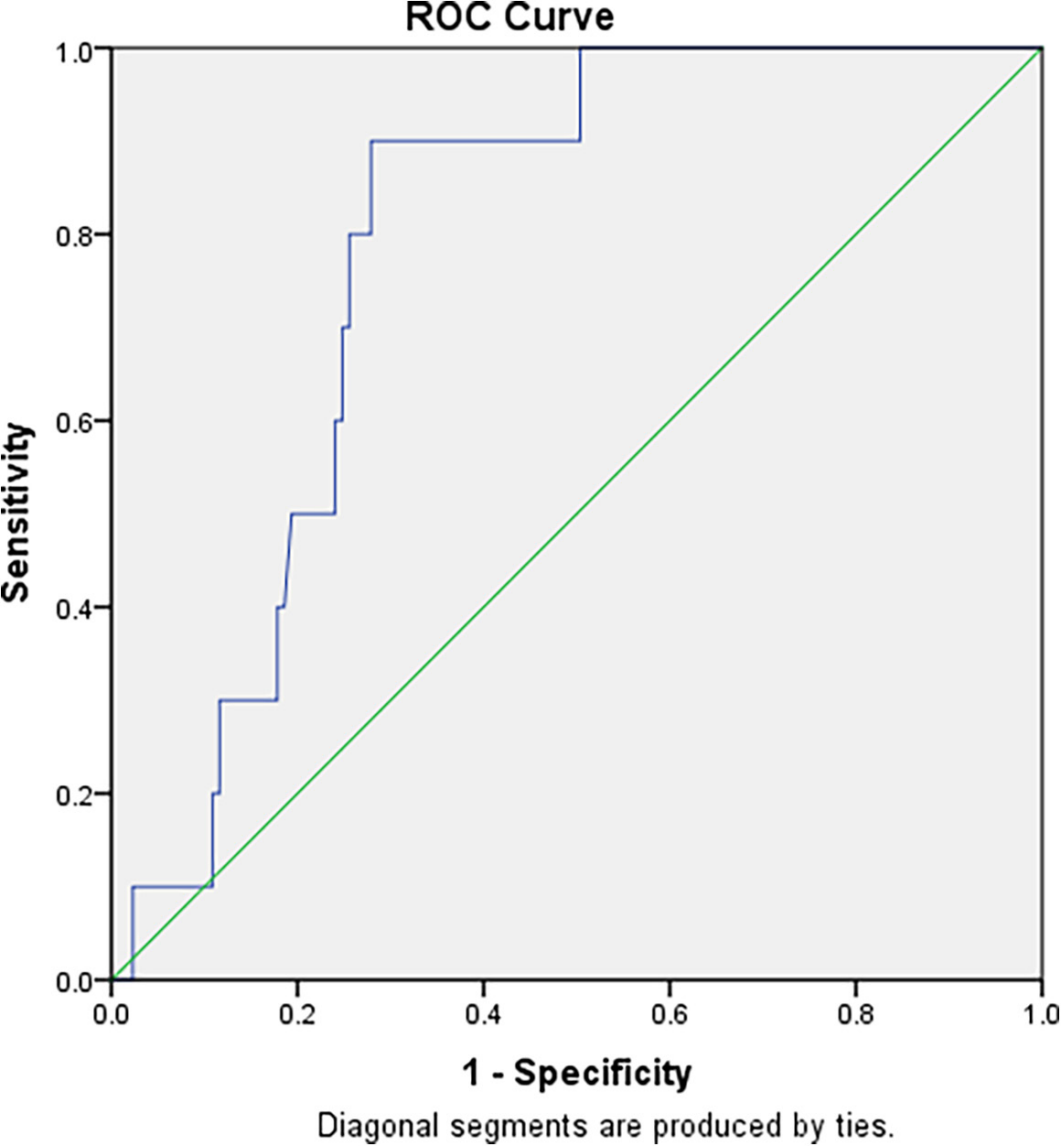
Random blood glucose on cardiovascular outpatient clinic visitation positively discriminates NOAF based on the ROC curve with area under the curve (AUC) 0.786 (*p* = .003) with the best cut‐off at 182 mg/dL (sensitivity 80% and specificity 72%). AUC, area under the curve; NOAF, new‐onset atrial fibrillation; ROC, receiving operator characteristic.

As can be seen in Table 3, random blood glucose level on visitation day was identified as an independent predictor of NOAF incidence in cardiovascular outpatient clinics. For every increasement of RBG by 1 unit, the risk of NOAF incidence raised 1.008 times (*p* = .034). Random blood glucose cut‐off to discriminate NOAF incidence was best found at 182 mg/dL, and can be seen at ROC curve in Figure 2.

## 4 DISCUSSION

The characteristics of subjects with HT or Diabetes Mellitus in predicting the risk factors of AF have previously been discussed by several other studies,^7^, ^8^, ^9^ but in this study to the best of our knowledge the addition of variables in the form of blood sugar cut‐off values, and heart valve abnormalities is the first. In this study, the characteristics of the subject's glucose level were similar to other studies which said that glycemic control had an effect on the risk of AF.^10^ There are still no studies that show the exact limit of glucose levels needed to reach a safe point where these levels can reduce the risk of NOAF.

From our result, we discovered that in hypertensive population, random blood glucose, along with valvular heart disease, was linked to a higher risk of NOAF as an independent predictor. It was similar to a prior study that found hypertensive people without ischemic vascular disease had a small association between diabetes and new‐onset atrial fibrillation.^11^ Another study found that hypertension's presence, severity, and length presence, severity, and length of hypertension were significant risk factors for new‐onset AF.^12^ Diabetes has been linked to an increased chance of having an arrhythmia (AF) in a number of groups, with the risk typically increasing by 10% to 30%. Diabetes might provide a unique pathophysiological substrate that, in theory, would exacerbate AF and make thromboembolic consequences worse. However, the ORBIT‐AF Registry study showed that there was no increased risk of AF development with diabetes.^13^ Furthermore, among patients with AF who live in the community, diabetes is independently linked to a higher risk of both all‐cause and cardiovascular mortality. Meanwhile, similar to our finding, previous studies also showed that valvular heart disease is associated with increased risk of AF.^14^, ^15^

Although the pathophysiological mechanisms causing AF are not fully known, a number of factors may play a role in its onset. Previous research demonstrated that hypertension, which affects 1 in 5 to 6 incidence occurrences of AF, is one of the main correctable causes.^16^, ^17^ The BP level and variability showed a value for predicting incident AF. In response to hypertension, left ventricular hypertrophy and decreased diastolic function cause an elevated left ventricular end‐diastolic pressure, which may raise left atrial pressure and volume, cause abnormal left atrial remodeling, and result in the development of AF. Despite earlier research showing a link between hypertension and AF, the dose‐response relationship of the hypertension burden as assessed by annual BP tests is still unclear. An earlier analysis found that the risk of AF and the burden of hypertension were both rising gradually. Additionally, the degree of elevated blood pressure plays a part in the onset of AF.^9^ However, in our result, degree of systolic blood pressure is not the predictor of new onset AF in this population. The difference in this result might be caused by the effect of the medication of hypertension treatment and difference in study design used in our study.

Meanwhile, epidemiology studies have demonstrated the connection between DM and AF. Although the exact cause of AF is still unknown, studies have suggested that inflammation may contribute to its development, maintenance, and perpetuation. There were pronounced inflammatory infiltrates and elevated CRP and IL‐6 in the atrial biopsies of patients with lone AF. In AF patients compared to the control group without atrial arrhythmia, CRP was two times greater. Additionally, it has been shown that the pathologic processes of DM may be caused by an inflammatory response. The myocardium in the atrium and ventricle can be directly impacted by glucose and insulin dysregulation, which can result in AF. Numerous epidemiology studies have linked poor glucose tolerance and left ventricular (LV) hypertrophy to diabetes mellitus (DM). A substantial risk factor for AF is LV hypertrophy. In addition, there was a strong correlation between LV mass and insulin resistance in both women with normal and poor glucose tolerance.^18^ Since LV hypertrophy is also found in HT, it can also explain the possible mechanism that connects hypertension, DM, and AF.

In several studies, it has been shown that elevated blood sugar levels are strongly associated with increased rates of cardiovascular events with a poor prognosis, but this relationship is not specific to arrhythmias and is rarely studied.^11^, ^19^ In this study, it was found that an increase in random blood sugar by 1 point would affect the risk of NOAF by 1.018× higher, and this is in accordance with a study that was conducted in Guangzhou, China which said that patients with elevated blood sugar would experience an increased risk of AF of 1.94 × is higher than normal.^20^

There are still no studies that show the exact limit of glucose levels needed to reach a safe point where these levels can reduce the risk of NOAF. From the results of this study, it was found that the glucose rate that could trigger an increase in the occurrence of AF was at ±186 mg/dL, this figure was in accordance with the results of a meta‐analysis study which said that at some point glucose level could lead to an increased risk of NOAF not only in diabetic (28%) state but also in pre‐diabetic (20%) state with an increase of 12%/20 mg/dL of blood sugar itself.^21^ In addition, other studies also state that impaired fasting glucose at pre‐diabetic levels is associated with the risk of AF as well as HF.^22^

There are some limitations in this study. This is a single‐center study, with a limited sample population. However, as far as we know, this is the first study that managed to make a target cut‐off for blood glucose level to decrease the risk of new‐onset AF incidence in HT and DM population; therefore, this study could act as a pilot study and the basis for further bigger cohort study in the future. From our result, we suggest a nationwide study with multicenter collaboration should be done next to prove our findings in the larger population.

## 5 CONCLUSION

This study was the first study that used random blood glucose level to predict the risk factors of newly diagnosed AF on the hypertension population DM comorbidity. Based on our result, it can be concluded that random blood glucose above 182 mg/dL and valvular heart disease can be used as a risk predictor of NOAF in HT and DM population, and highlighted the importance of blood glucose control in this population. Further study is needed to validate this finding in a larger population.

## CONFLICT OF INTEREST STATEMENT

The authors declare that the research was conducted in the absence of any commercial or financial relationships that could be considered as a potential conflict of interests.

## ETHICS STATEMENT

Since our study used secondary data and was an observational analytical study, no ethical approval was required.

## Supporting information


Appendix S1.

## Data Availability

The data that support the findings of this study are available on request to the corresponding author.

## References

[1] Hamdan H , Tatisina CM . Pelaksanaan Pemberdayaan Keluarga Dan Senam Hipertensi Sebagai Upaya Manajemen Diri Penderita Hipertensi. Jurnal Pengamas Kesehatan Sasambo. 2020;1(2):75–79.

[2] Sugiarta GRM , Darmita GK . Profil penderita Diabetes Mellitus Tipe‐2 (DM‐2) Dengan Komplikasi Yang Menjalani Rawat Inap di Rumah Sakit Umum Daerah (RSUD) Klungkung, Bali tahun 2018. Intisari Sains Medis. 2020;11(1):7–12.

[3] Tatsumi Y , Ohkubo T . Hypertension with diabetes mellitus: significance from an epidemiological perspective for Japanese. Hypertens Res. 2017;40(9):795–806. 28701739 10.1038/hr.2017.67

[4] Okin PM , Hille DA , Kjeldsen SE , Devereux RB . Combining ECG criteria for left ventricular hypertrophy improves risk prediction in patients with hypertension. J Am Heart Assoc. 2021;6(11):e007564. 10.1161/JAHA.117.007564 PMC5721804 29151037

[5] Liu CY , Zhang W , Ji LN , Wang JG , ATTEND Investigators . Comparison between newly diagnosed hypertension in diabetes and newly diagnosed diabetes in hypertension. Diabetol Metab Syndr. 2019;11(1):69. 31462932 10.1186/s13098-019-0465-3 PMC6708242

[6] Kelsey JL , Whittemore AS , Evans AS , Thompson WD . Methods in observational epidemiology. Oxford University Press; 1996.

[7] Coccina F , Pierdomenico AM , Ianni U , De Rosa M , De Luca A , Pirro D , et al. Ambulatory blood pressure and risk of new‐onset atrial fibrillation in treated hypertensive patients. J Clin Hypertens. 2021;23(1):147–152. 10.1111/jch.14112 PMC8029687 33242233

[8] Fatemi O , Yuriditsky E , Tsioufis C , Tsachris D , Morgan T , Basile J , et al. Impact of intensive glycemic control on the incidence of atrial fibrillation and associated cardiovascular outcomes in patients with type 2 diabetes mellitus (from the Action to Control Cardiovascular Risk in Diabetes Study). Am J Cardiol. 2014;114(8):1217–1222. 25159234 10.1016/j.amjcard.2014.07.045 PMC4291278

[9] Lee SR , Park CS , Choi EK , Ahn HJ , Han KD , Oh S , et al. Hypertension burden and the risk of new‐onset atrial fibrillation: a nationwide population‐based study. Hypertension. 2021;77(3):919–928. 33486985 10.1161/HYPERTENSIONAHA.120.16659

[10] Dublin S , Glazer NL , Smith NL , Psaty BM , Lumley T , Wiggins KL , et al. Diabetes mellitus, glycemic control, and risk of atrial fibrillation. J Gen Intern Med. 2010;25(8):853–858. 20405332 10.1007/s11606-010-1340-y PMC2896589

[11] Alves‐Cabratosa L , García‐Gil M , Comas‐Cufí M , Martí R , Ponjoan A , Parramon D , et al. Diabetes and new‐onset atrial fibrillation in a hypertensive population. Ann Med. 2016;48(3):119–127. 26939743 10.3109/07853890.2016.1144930

[12] Kim YG , Han KD , Choi JI , Yung Boo K , Kim DY , Oh SK , et al. Impact of the duration and degree of hypertension and body weight on new‐onset atrial fibrillation. Hypertension. 2019;74(5):e45–e51. 31522617 10.1161/HYPERTENSIONAHA.119.13672

[13] Echouffo‐Tcheugui JB , Shrader P , Thomas L , Gersh BJ , Kowey PR , Mahaffey KW , et al. Care patterns and outcomes in atrial fibrillation patients with and without diabetes: ORBIT‐AF registry. J Am Coll Cardiol. 2017;70(11):1325–1335. 28882229 10.1016/j.jacc.2017.07.755

[14] Lip GYH , Collet JP , de Caterina R , Fauchier L , Lane DA , Larsen TB , et al. Antithrombotic therapy in atrial fibrillation associated with valvular heart disease: a joint consensus document from the European heart rhythm association (EHRA) and European Society of Cardiology Working Group on thrombosis, endorsed by the ESC working group on Valvular heart disease, cardiac arrhythmia Society of Southern Africa (CASSA), Heart Rhythm Society (HRS), Asia Pacific Heart Rhythm Society (APHRS), south African heart (SA heart) association and Sociedad Latinoamericana de Estimulación Cardíaca y Electrofisiología (SOLEACE). EP Europace. 2017;19(11):1757–1758. 29096024 10.1093/europace/eux240

[15] Bhardwaj R . Atrial fibrillation in a tertiary care institute ‐ a prospective study. Indian Heart J. 2012;64(5):476–478. 23102385 10.1016/j.ihj.2012.07.014 PMC3861218

[16] Benjamin EJ , Levy D , Vaziri SM , D'Agostino RB , Belanger AJ , Wolf PA . Independent risk factors for atrial fibrillation in a population‐based cohort: the Framingham heart study. JAMA. 1994;271(11):840–844. 8114238

[17] Gorenek B , Pelliccia A , Benjamin EJ , Boriani G , Crijns HJ , Fogel RI , et al. European Heart Rhythm Association (EHRA)/European Association of Cardiovascular Prevention and Rehabilitation (EACPR) position paper on how to prevent atrial fibrillation endorsed by the Heart Rhythm Society (HRS) and Asia Pacific Heart Rhythm Society (APHRS). Europace. 2017;19(2):190–225. 28175283 10.1093/europace/euw242 PMC6279109

[18] Sun Y , Hu D . The link between diabetes and atrial fibrillation: cause or correlation? J Cardiovasc Dis Res. 2010;1(1):10–11. 21188083 10.4103/0975-3583.59978 PMC3004163

[19] Hsu JC , Yang YY , Chuang SL , Yu CC , Lin LY . Higher long‐term visit‐to‐visit glycemic variability predicts new‐onset atrial fibrillation in patients with diabetes mellitus. Cardiovasc Diabetol. 2021;20(1):148. 34301257 10.1186/s12933-021-01341-3 PMC8305511

[20] Fu L , Deng H , Lin WD , He SF , Liu FZ , Liu Y , et al. Association between elevated blood glucose level and non‐valvular atrial fibrillation: a report from the Guangzhou heart study. BMC Cardiovasc Disord. 2019;19(1):270. 31779588 10.1186/s12872-019-1253-6 PMC6883689

[21] Aune D , Feng T , Schlesinger S , Janszky I , Norat T , Riboli E . Diabetes mellitus, blood glucose and the risk of atrial fibrillation: a systematic review and meta‐analysis of cohort studies. J Diabetes Complications. 2018;32(5):501–511. 29653902 10.1016/j.jdiacomp.2018.02.004

[22] Lind V , Hammar N , Lundman P , Friberg L , Talbäck M , Walldius G , et al. Impaired fasting glucose: a risk factor for atrial fibrillation and heart failure. Cardiovasc Diabetol. 2021;20(1):227. 34819087 10.1186/s12933-021-01422-3 PMC8614025

