# Supplementary material for: ECG abnormality and predictor of new‐onset atrial fibrillation in hypertension and diabetes mellitus population: An observational analytic study from cardiovascular outpatient clinic at a National Cardiovascular Center in Indonesia
Source: J Arrhythm. 2023 Oct 9;39(6):860–7. doi: 10.1002/joa3.12938 (PMC10692857; doi:10.1002/joa3.12938)
Supplement: Supplementary file 1 — Appendix S1. [file JOA3-39-860-s001.docx]

Supplementary data

Table 1. Definition used to determine ECG abnormality.

| **ECG Abnormality** | **Definition Used** |
| --- | --- |
| Left bundle branch block | 1) QRS duration greater than or equal to 120 ms, 2) Broad notched or slurred R wave in leads I, aVL, V5, and V6 and an occasional RS pattern in V5 and V6 attributed to displaced transition of QRS complex, 3) Absent q waves in leads I, V5, and V6, but in the lead aVL, a narrow q wave may be present in the absence of myocardial pathology, 4) R peak time greater than 60 ms in leads V5and V6but normal in leads V1, V2, and V3, when small initial r waves can be discerned in the above leads, 5) ST and T waves usually opposite in direction to QRS, 6) Positive T wave in leads with upright QRS may be normal (positive concordance), 7) Depressed ST segment and/or negative T wave in leads with negative QRS (negative concordance) are abnormal and are discussed in part VI of this statement, 8) The appearance of LBBB may change the mean QRS axis in the frontal plane to the right, to the left, or to a superior, in some cases in a rate-dependent manner (1) |
| Right bundle branch block | 1) QRS duration was greater than or equal to 120 ms, 2) rsr′, rsR′, or rSR′ in leads V1or V2. The R′ or r′ deflection is usually wider than the initial R wave. In a minority of patients, a wide and often notched R wave pattern may be seen in lead V1and/or V2. S wave of greater duration than R wave or greater than 40 ms in leads I and V6. Normal R peak time in leads V5 and V6 but greater than 50 ms in lead V1 (1) |
| Left atrial enlargement | any one of the following: 1) P wave in any lead > 0.11s, 2) Notched P wave with interpeak duration > 0.04s (P mitrale), 3) P wave axis < 30°, 4) Area of negative P terminal force in lead V1 (NPTF-V1) > 0.04s·mm, or 5) Positive P terminal force in aVL (PPTF-aVL) > 0.5 mm (2) |
| left ventricular hypertrophy | Sokolow-Lyon voltage criteria: the amplitude of the S wave in lead V1 was added to the largest amplitude of the R wave in either lead V5 or V6, with a value greater than or equal to 35 mm meeting criteria for LVH [42] |
| Right atrial enlargement | Any one of the following: 1) P wave in inferior leads II, III, aVF > 2.5 mm or 2) Positive P wave in V1 > 1.5 mm (2) |
| Right ventricular hypertrophy | Myers criteria, any one of the following: Tall R V1 > 6 mV, increased R:S ratio V1 > 1.0, Deep S V5 > 10 mV, Deep S V6 > 3 mV, Small S V1 < 2 mV, Small R V5,6 < 3 mV, Reduced R:S ratio V5 < 0.75, Reduced R:S ratio V6 < 0.4, R peak V1 (QRS duration<0.12 sec) > 0.035, Presence of QR V1 (3) |
| Pathologic Q wave | any Q wave with more than 40 ms width or a depth more than one-third of the adjacent R wave in more than two adjacent leads (4) |
| New onset atrial fibrillation (NOAF) | New onset or a first detectable episode of AF, whether symptomatic or not. Atrial fibrillation (AF) is a supraventricular tachyarrhythmia with uncoordinated atrial electrical activation and consequently ineffective atrial contraction. Electrocardiographic characteristics include: Irregularly irregular R-R intervals (where atrioventricular conduction is not impaired), Absence of distinct repeating P wave, and Irregular atrial activations. (5) |
| Premature atrial contraction (PAC) | 1) Premature atrial complex, occurring earlier than expected if measured against previous P-P intervals. 2) Ectopic P wave with different morphology with normal sinus, originating outside of the SA node 3) Narrow QRS complexes 4) Complete Compensatory pause following the contraction. (6) |
| Premature ventricular contraction (PVC) | Premature occurrence of an abnormal QRS complex (duration typically ≥120 ms, corresponding T-wave typically broad and in the opposite direction of the major QRS deflection, no preceding P-wave), either unifocal or multifocal.(7) |

1. Surawicz B, Childers R, Deal BJ, Gettes LS. AHA/ACCF/HRS Recommendations for the Standardization and Interpretation of the Electrocardiogram. Circulation. 2009 Mar;119(10):e235–40.

2. Mulia EPB, Nugraha RA, A’yun MQ, Juwita RR, Yofrido FM, Julario R, et al. Electrocardiographic abnormalities among late-stage non-dialysis chronic kidney disease patients. J Basic Clin Physiol Pharmacol [Internet]. 2021;32(3):155–62. Available from: <https://doi.org/10.1515/jbcpp-2020-0068>

3. Whitman IR, Patel V v, Soliman EZ, Bluemke DA, Praestgaard A, Jain A, et al. Validity of the surface electrocardiogram criteria for right ventricular hypertrophy: the MESA-RV Study (Multi-Ethnic Study of Atherosclerosis-Right Ventricle). J Am Coll Cardiol. 2013/09/28. 2014 Feb;63(7):672–81.

4. Arjmand A, Eshraghi A, Sani Z, Firouzi A, Sanati H, Nezami H, et al. Value of pathologic Q wave in surface electrocardiography in the prediction of myocardial nonviability: A cardiac magnetic resonance imaging-based study. J Adv Pharm Technol Res. 2018 Oct;9(4):162–4.

5. Hindricks G, Potpara T, Dagres N, Arbelo E, Bax JJ, Blomström-Lundqvist C, et al. 2020 ESC Guidelines for the diagnosis and management of atrial fibrillation  developed in collaboration with the European Association for Cardio-Thoracic Surgery (EACTS): The Task Force for the diagnosis and management of atrial fibrillation of the Europe. Eur Heart J. 2021 Feb;42(5):373–498.

6. Surawicz B, Knilans T. Chou’s Electrocardiography in Clinical Practice: Adult and Pediatric, Sixth Edition. Sixth, editor. Saunders, Philadelphia; 2008.

7. Zeppenfeld K, Tfelt-Hansen J, de Riva M, Winkel BG, Behr ER, Blom NA, et al. 2022 ESC Guidelines for the management of patients with ventricular arrhythmias and the prevention of sudden cardiac death: Developed by the task force for the management of patients with ventricular arrhythmias and the prevention of sudden cardiac death of the European Society of Cardiology (ESC) Endorsed by the Association for European Paediatric and Congenital Cardiology (AEPC). European Heart Journal. 2022 Oct 21;43(40):3997–4126.
